# Role of magnetic resonance diffusion imaging and apparent diffusion coefficient values in the evaluation of spinal tuberculosis in Indian patients

**DOI:** 10.4103/0971-3026.73544

**Published:** 2010-11

**Authors:** Lalitha Palle, MCH Balaji Reddy, K Jagannath Reddy

**Affiliations:** Department of Radiology, Focus Diagnostics, Hyderabad, Andhra Pradesh, India

**Keywords:** Diffusion MRI, spine, tuberculosis

## Abstract

**Aim:**

To define a range of apparent diffusion coefficient values in spinal tuberculosis and to evaluate the sensitivity of diffusion-weighted magnetic resonance imaging (DW-MRI) and apparent diffusion coefficient values in patients of spinal tuberculosis.

**Materials and Methods:**

This study was conducted over a period of 20 months and included 110 patients with a total of 230 vertebral bodies. The study was performed in two parts. The first part included all patients of known tuberculosis and patients with classical features of tuberculosis. The second part included patients with spinal pathology of indeterminate etiology. All the patients underwent a routine MRI examination along with diffusion sequences. The apparent diffusion coefficient (ADC) values were calculated from all the involved vertebral bodies.

**Results:**

The mean ADC value of affected vertebrae in first part of the study was found to be 1.4 ± 0.20 × 10^−3^ mm^2^/s. This ADC value was then applied to patients in the second part of study in order to determine its ability in predicting tuberculosis. This range of ADC values was significantly different from the mean ADC values of normal vertebrae and those with metastatic involvement. However, there was an overlap of ADC values in a few tuberculous vertebrae with the ADC values in metastatic vertebrae.

**Conclusion:**

We found that DW-MRI and ADC values may help in the differentiation of spinal tuberculosis from other lesions of similar appearance. However, an overlap of ADC values was noted with those of metastatic vertebrae. Therefore diffusion imaging and ADC values must always be interpreted in association with clinical history and routine MRI findings and not in isolation.

## Introduction

Spinal tuberculosis (TB), also called Pott’s spine, is quite common in India. It can be confidently diagnosed on MRI if there is involvement of contiguous vertebrae and intervening discs, with extensive adjacent soft tissue involvement and abscess formation. It can sometimes be difficult to diagnose this condition with confidence in the very early stages, especially when there is isolated vertebral body involvement without any soft tissue component, adjacent disc involvement, or abscess formation. In this prospective study, we aim to quantify and evaluate the role of diffusion MRI and apparent diffusion coefficient (ADC) values in tuberculous vertebrae. Diffusion-weighted MRI is now used extensively and has an established role in brain imaging. It is based on the principle of Fick’s law of concentration gradients and Brownian movement of molecules within a given tissue. ADC values are a measure of the diffusion ability of molecules in the given tissue and give an idea of the composition of the given tissue.[1] A high ADC value means increased Brownian movement of molecules (which means no restriction), thereby suggesting less compactness of the given tissue microstructure. Many studies have been performed till now and the role of DW-MRI in metastatic spinal involvement has been established. To the best of our knowledge, there are no previous studies that have exclusively tried to establish ADC values in cases of spine TB.

Since spine TB is so extensive in our country, we have tried to quantify and evaluate the role of DW-MRI and ADC values in this condition, which we hope will increase diagnostic confidence in doubtful cases and decrease the need for biopsy.

## Materials and Methods

This study was conducted in two parts. In the first part, 56 patients with 128 abnormal vertebrae, either known to have tuberculosis or with classic tuberculous findings were imaged with DW-MRI.

In the second part (54 patients), the ADC value that was arrived from the first part was used to predict its usefulness in diagnosing tuberculosis in 102 vertebrae with indeterminate pathology.

There were 70 males and 40 female patients, with the ages ranging from 12 to 90 years. Informed consent was obtained from all the patients. Detailed clinical history of all patients was taken before the start of the examination. Inclusion and exclusion criteria were as follows:

### Inclusion criteria

1. Known cases of spinal tuberculosis and those with classical features (subset 1). This subset included 128 vertebrae.
2. Patients with spinal involvement but who were not proven cases of tuberculosis and who did not have the classical appearance of either tuberculosis or metastasis, irrespective of the ADC values. (This subset of patients was followed up till a diagnosis was established. They did not have any significant past history of trauma, osteoporosis, or malignancy) (subset 2). This subset included 102 vertebrae.

### Exclusion criteria

1. Patients with spine involvement due to trauma, osteoporotic collapse, metastasis, or any known disease other than tuberculosis.
2. Patients without any follow-up or those lost to follow-up.

All patients underwent a routine plain MRI of the spine. DW-MRI was also performed in the same sitting. MRI of the spine, along with DW-MRI, was performed on a 1.5-T Siemens Avanto machine. The MRI protocol included T1 sagittal (TR – 430 ms, TE – 60 ms); T2 sagittal and axial (TR – 4200 ms, TE – 120 ms); and STIR coronal and sagittal (TR – 4000 ms, TE – 70 ms). Slice thickness was 3 mm. Field of view (FOV) was 32–37 cm.

The MRI pulse sequence used for DW-MRI was a single-shot echo-planar sequence. Sagittal diffusion imaging was performed at TR – 2400 ms, TE – 88 ms, slice thickness – 4 mm, FOV – 35 × 35 cm, number of excitations – 1, and matrix – 128 × 128. Sagittal DW-MRI was performed at b values of 0, 500, and 1000. ADC values were measured on the sagittal images, as localization and placement of region of interest (ROI) was easier on the sagittal than axial images. ADC values were automatically computed after placing the ROI cursor entirely within the area of abnormal vertebral body signal intensity. Care was taken to place ROIs of same size and to see that there was no overlap with the adjacent disc or soft tissues. The smallest available ROI of 0.03 cm^2^ was used. The scanner software provides the mean value within the ROI, which equals the ADC value (multiplied by 10^−3^). Two normal-appearing vertebral bodies adjacent to the affected vertebrae were also sampled and the ADC values recorded. The final mean ADC value in all the proven cases of tuberculosis was calculated.

## Results

**Figure 1 (A–E):**
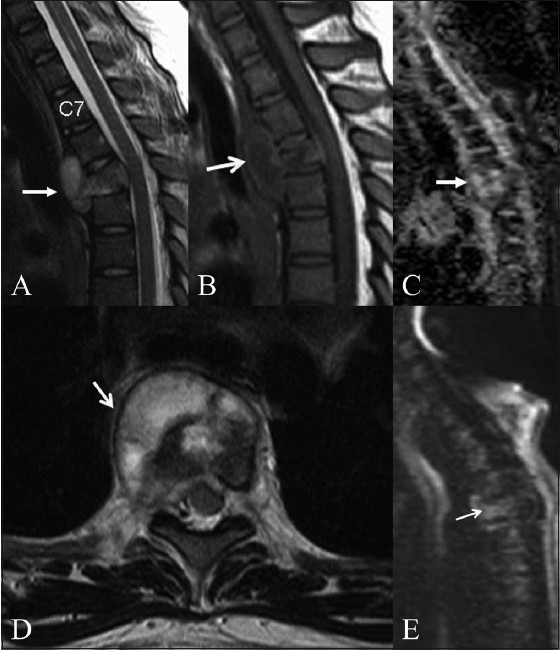
Tuberculosis of the spine. T2W (A), T1W (B) axial MRI images in a 12-year-old girl with upper dorsal spine tuberculosis show a small prevertebral abscess (arrow). Wedging of the D2 vertebral body is seen with marrow involvement. Sagittal ADC map (C) sagittal and T2W (D) and diffusion (E) images show increased diffusion (arrow) in the involved vertebra (ADC: 1.35 × 10^−3^ mm^2^/s).

**Figure 2 (A–E):**
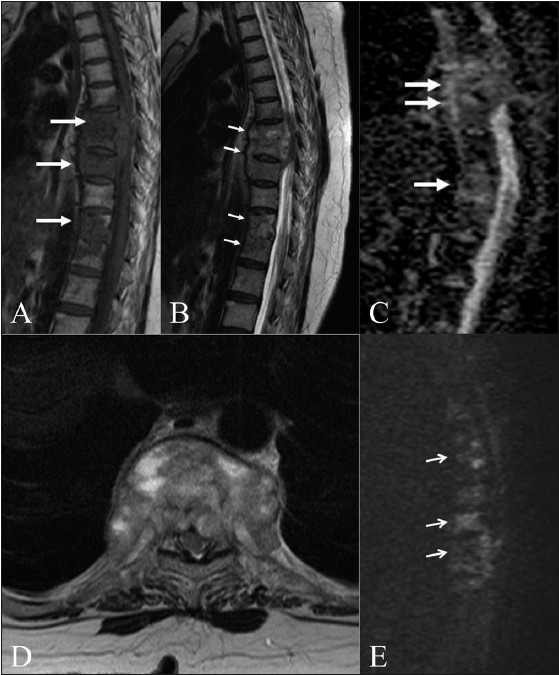
Tuberculosis of the spine. Sagittal T1W (A) and T2W (B) images in an elderly patient with tuberculosis show multifocal dorsal vertebral body involvement (arrows) with an epidural soft tissue component. Sagittal ADC map (C) and axial T2W (D) and diffusion (E) images show increased diffusion (arrows) in the involved vertebrae. (ADC: 1.42–1.5 × 10^−3^ mm^2^/s)

**Figure 3 (A–C):**
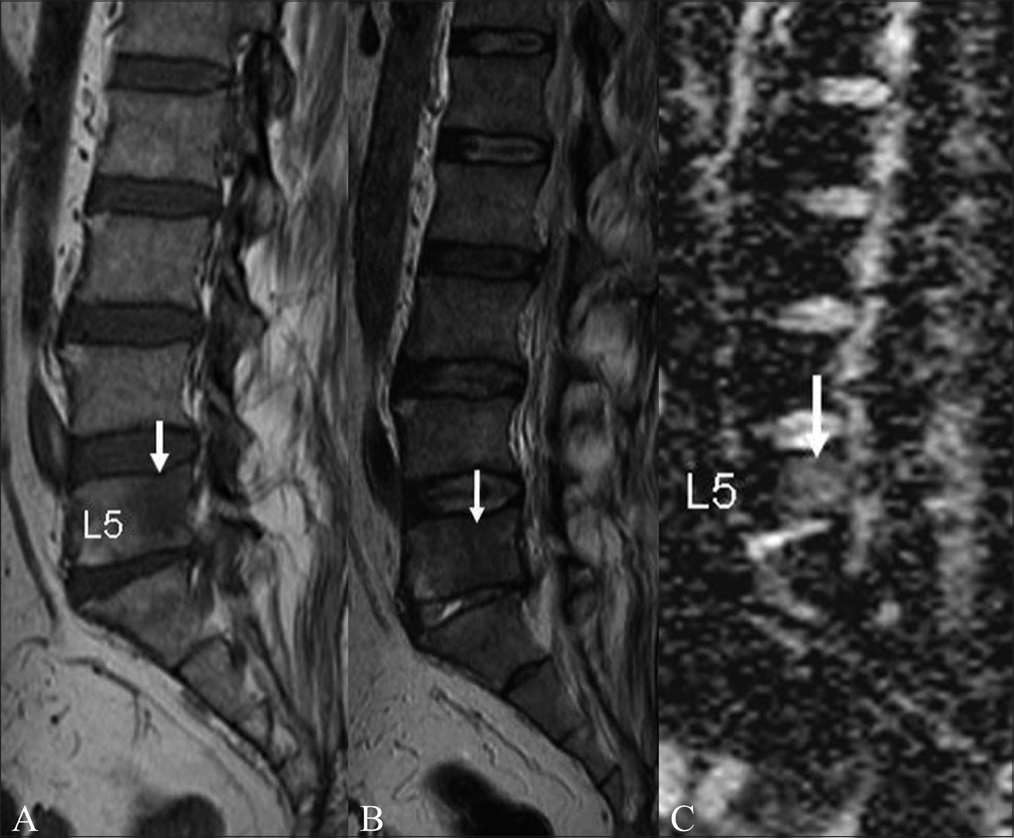
Tuberculosis of spine. Sagittal T1W (A) and T2W (B) images in a 40-year-old patient with spinal tuberculosis show a well-defined rounded lesion (arrow) with T1 hypointensity and T2 iso-hypointensity, which was difficult to characterize on routine MRI imaging. Sagittal ADC map (C) reveals increased diffusion in the lesion with an ADC value of 1.46 × 10^−3^ mm^2^/s

**Figure 4 (A–F):**
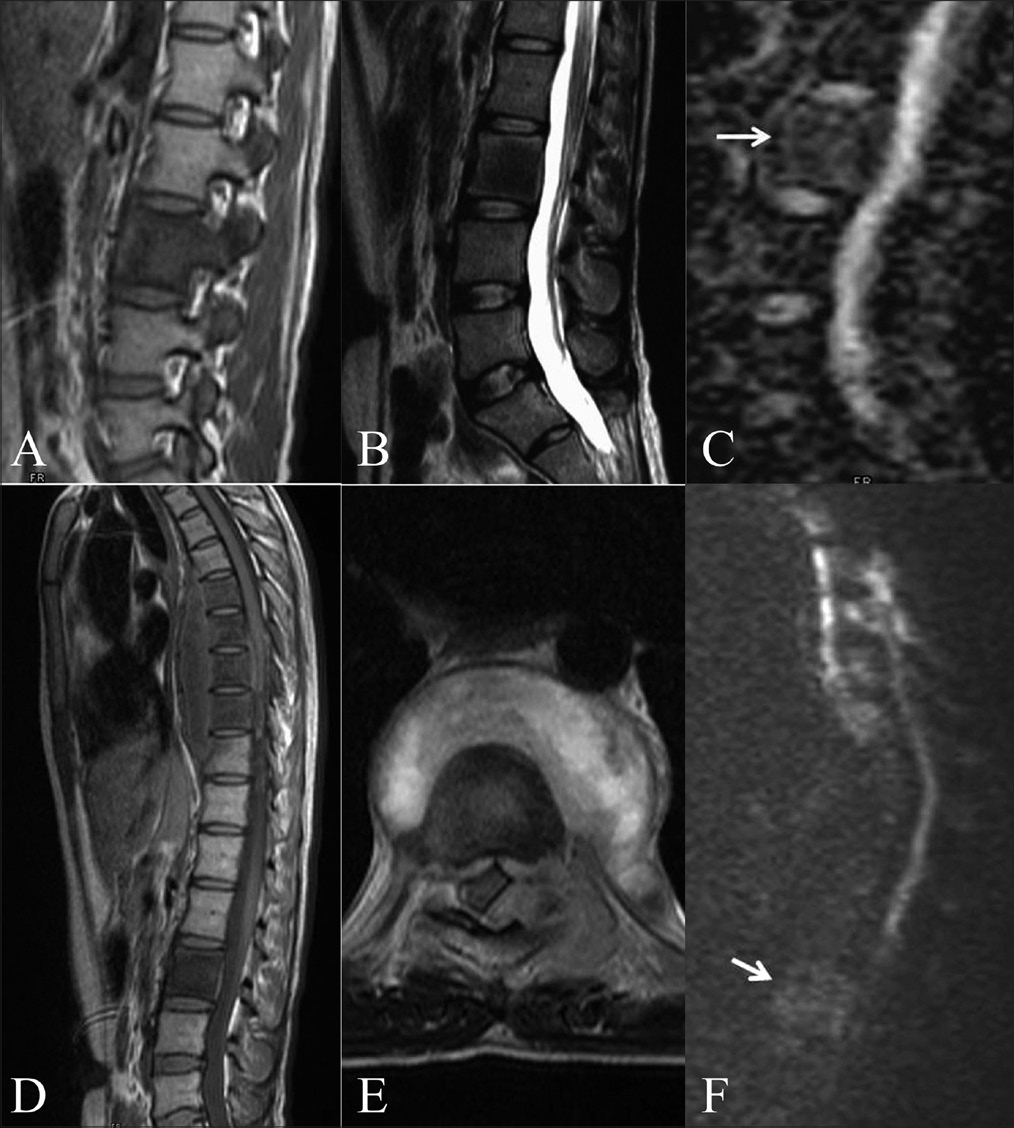
Tuberculosis initially misdiagnosed as metastasis. Parasagittal T1W (A) and sagittal T2W (B) images show a lesion with T1 and T2 hypointensity in the L1 vertebra and pedicle. Sagittal ADC map (C) reveals mild restriction of diffusion with an ADC value of 0.90 × 10^−3^ mm^2^/s. Sagittal T1W (D) and axial T2W (E) images after 6 months reveal multifocal lesions with T1 hypointensity, from D5 to D8, with a prevertebral abscess and an epidural soft tissue component. Sagittal diffusion image (F) at this time, reveals mildly bright signals in the involved dorsal vertebrae. The previously effected L1 vertebra (arrow in F) shows no significant change

In the first part of the study, of the 128 vertebral bodies, 10 were in the cervical spine, 57 were in the dorsal spine [Figures 1A–E, 2A–E], and the remaining 61 were in the lumbar spine [Figure 3A–C]. Fourteen patients had solitary vertebral body involvement, while the rest had involvement of multiple vertebrae. Forty percent of patients had pre and paravertebral soft tissue involvement of varying degrees. Psoas abscesses were noted in 25% of patients. Four patients had involvement of posterior elements [Figure 4A–F].

**Figure 5 (A, B):**
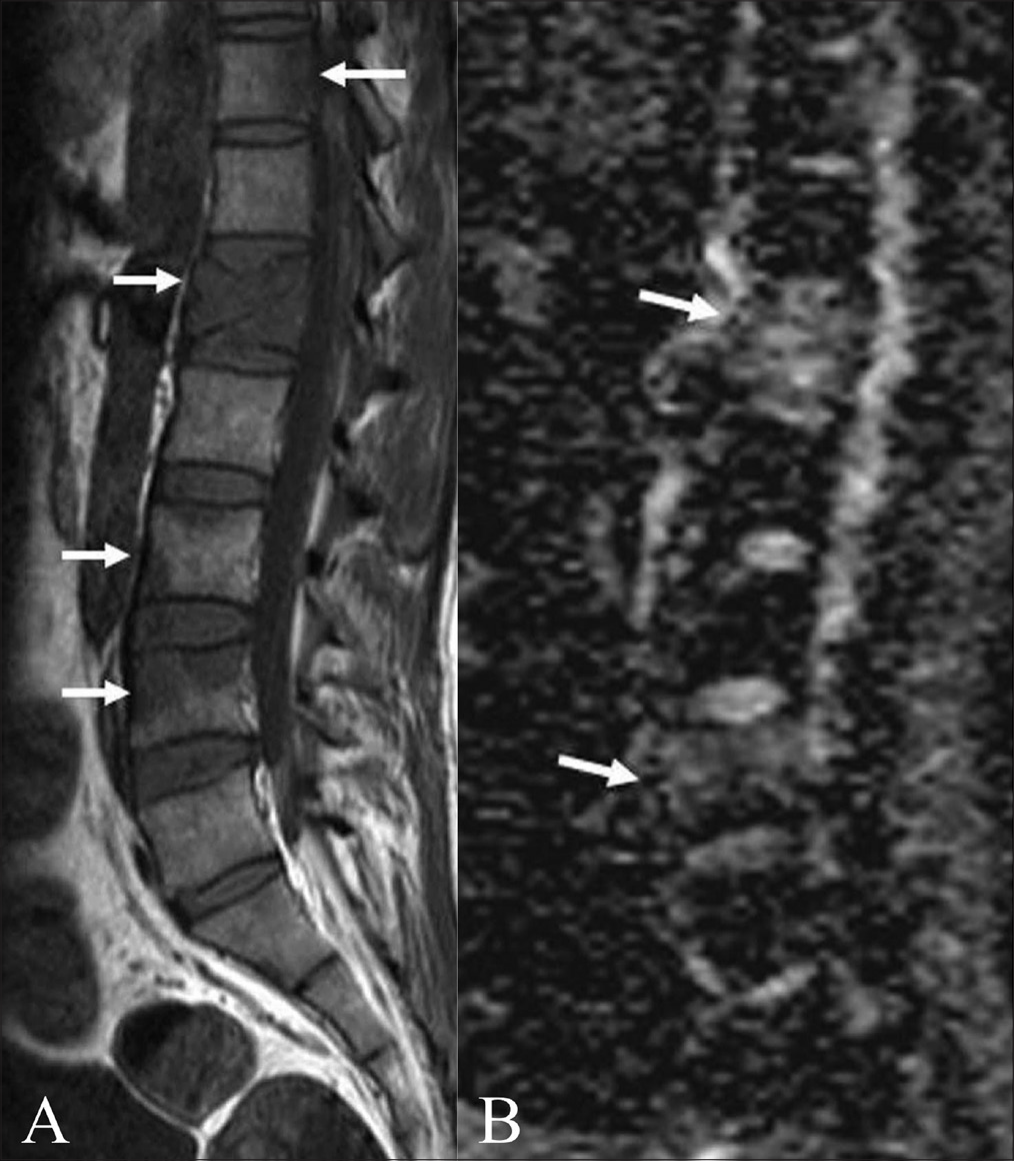
Tuberculosis of the spine. Sagittal T1W image of a 35-year-old patient shows multiple vertebral body lesions (arrows) and wedging of L1, with no significant soft tissue component. It was not possible to differentiate between tuberculosis and metastases. Sagittal ADC map (B) reveals increased diffusion in the lesions, with ADC values of 1.42–1.49 × 10^−3^ mm^2^/s, suggesting that metastatic involvement was probably less likely

All the involved vertebrae showed T1 hypointense signals [Figure 5A, B] with STIR hyperintensity. The T2 appearance was variable, ranging from hypointense to hyperintense signals. The vertebrae (82) with T2 hyperintense signals revealed high ADC values and appeared brighter on the ADC images, while the vertebrae with T2 hypo/isointense signals (46) revealed relatively lower ADC values and appeared less bright/dark on ADC images. The mean ADC value was 1.4 ± 0.20 × 10^−3^ mm^2^/s.

This ADC value was then applied to determine its ability to predict tuberculosis in the second part of the study in 102 vertebrae.

Out of the 102 vertebrae in the second part of the study, 48 were finally proven to be cases of metastatic involvement and the remaining 54 were proven to be of tuberculous etiology [Table 1]. Based on the ADC values obtained in the first part of the study, we falsely reported 12 as tuberculous vertebrae based on high ADC values, which were later on proven to be metastatic. We also reported 19 of the finally proven 54 tuberculous vertebrae as metastatic lesions in view of the low ADC values. In total there were 47 vertebrae with high ADC values and 55 vertebrae with low ADC values. Based on all the results obtained we finally calculated that the mean ADC value of 1.4 ± 0.20 × 10^−3^ mm^2^/s had a sensitivity of 64.8% and specificity of 75% for predicting spinal tuberculosis. The positive predictive value was 74.5%. Using Fisher’s exact test, the *p* value was calculated and found to be significant. (*p* < 0.0001).

**Table 1:** Results

Study	Subset 1	Subset 2
Patients	Includes patients of known TB and those with classical findings of TB	Includes patients of spinal pathology of indeterminate etiology (Refer to Inclusion criteria)
Number	56 patients and 128 vertebrae	54 patients and 102 vertebrae
Location	Cervical – 10, Dorsal – 57, Lumbar – 61	Cervical – 9, Dorsal – 52, Lumbar – 41
Final diagnosis	All were TB vertebrae	Out of 102 vertebrae, 48 were finally metastatic and 54 were finally TB.
ADC values	Mean – 1.4 ± 0.20 × 10^−3^ mm^2^/s	12 metastatic vertebrae reported as TB vertebrae (False +ve) had ADC values from 1.2 to 1.40 × 10^−3^ mm^2^/s
		19 TB vertebrae reported as metastatic had ADC values from (False −ve) - 0.86 to 1.0 × 10^−3^ mm^2^/s
		35 TB vertebrae (True +ve) - 1.4 ± 0.20 × 10^−3^ mm^2^/s
		36 metastatic vertebrae (True −ve) - 0.83 to 1.0 × 10^−3^ mm^2^/s

## Discussion

DW-MRI has been extensively studied and used in the imaging of brain. It provides information about the composition of tissues, physical properties, and the microstructure of the tissues.[1] DW-MRI performed at high b value (1000) is a sensitive predictor of water mobility within the tissues and reduces the phenomenon of T2 shine-through. To the best of our knowledge, there are no previous studies that have exclusively focused on evaluating DW-MRI in spine TB. Many studies have been performed in different parts of the world to evaluate the efficacy of DW-MRI in differentiating between malignant and benign vertebral body involvement; in these studies the benign lesions included infections and traumatic osteoporotic vertebral body involvement.[2–4] All these studies have found that the ADC values of metastatic vertebral bodies are significantly different than those of benign lesions.[2–4]

The presence of pre/paravertebral abscess and the involvement of contiguous vertebrae and intervening discs make the diagnosis of spine TB quite obvious. In cases where these classical features of spine TB are not present, there may be some difficulty in differentiating tuberculous lesions from malignant infiltration. In such cases, DW-MRI can prove to be a useful tool to arrive at the correct diagnosis.

There is more free water content in the marrow in benign vertebral body involvement as compared to malignant infiltration.[2] In malignant infiltration, there is dense compact tumor infiltration, which inhibits free movement of water molecules, thereby causing diffusion restriction.[2] The areas of restricted diffusion appear bright on DW-MRI and dark on ADC mapping.

The initial studies on DW-MRI of vertebral bodies were conducted by Beaur *et al*.[3] and Spuenturp *et al*.[4] Both the studies showed promising results. However, their studies were conducted on a small patient population (30 patients in the study by Beaur *et al*. and 34 patients in the one by Spuenturp *et al*.) and the b values were also lower (b = 165 s/mm ^2^ in the Boeur *et al*. study and b = 598 s/mm^2^ in the Spuenturp *et al*. study).

The study by Maeda *et al*. included 64 patients; the b value in their study was 1000 cm/mm^2^.[5] In their study, there was an overlap between the ADC values of benign and malignant vertebral body disease; however, the ADC values of benign lesions were significantly higher than those of malignant lesions. The mean ADC in benign vertebral body compression fractures was 1.21 ± 0.17 × 10^−3^ mm^2^/s and in malignant vertebral compression fractures it was 0.92 ± 0.20 × 10^−3^ mm ^2^/s.[5] In the study conducted by Bhugaloo *et al*.[6] on 35 patients with 68 vertebral compression fractures, the positive and negative predictive values of diffusion MRI for detecting malignant vertebral body involvement were both 90%. In the study conducted by Herneth *et al*.[7] on 22 patients, the ADC value of vertebral metastases was 0.69 × 10^−3^ mm^2^/s, which is lower than that in other studies.

In our study, 56 patients with 128 tuberculous vertebrae had a mean ADC value of 1.4 ± 0.20 × 10 ^−3^ mm^2^/s. When this value was applied to vertebrae of indeterminate etiology, the sensitivity of this cut-off was 64.8%, the specificity, 75%, and the positive predictive value, 74.5%.

All the involved vertebrae in both parts of the study showed T1 hypointense and STIR hyperintense signals. In the first part of the study there were 46 vertebrae that showed T2 hypointense signals and 82 vertebrae with T2 hyperintense signals. T2 iso-hypointense signals were probably due to dense compact caseation in the vertebrae, while T2 hyperintense signals could be the result of liquefaction. The mean ADC value in all vertebrae showing T2 hypointense signals was 1.2 × 10^−3^ mm^2^ /s, while in those showing T2 hyperintense signals, it was 1.6 × 10 ^−3^ mm^2^ /s. In the second part of the study (Subset 2), there were 59 vertebrae with T2 iso-hypointense signals while rest of 43 vertebrae showed T2 hyperintense signals. All the involved vertebrae in both parts of the study revealed variable degrees of STIR hyperintensities.

## Conclusion

Diffusion-weighted magnetic resonance imaging may prove to be a relatively useful adjunct to conventional MRI in differentiating tuberculous vertebral body involvement from metastatic lesions. False negative results can be obtained when there is dense solid caseation within the vertebrae, and in this situation overlap with ADC values of malignant lesions may be noted. Therefore, diffusion MRI and ADC coefficient values are always best interpreted along with routine MRI sequences and a detailed clinical history and examination.
